# Evaluating Roles of Nodes in Optimal Allocation of Vaccines with Economic Considerations

**DOI:** 10.1371/journal.pone.0070793

**Published:** 2013-08-14

**Authors:** Bing Wang, Hideyuki Suzuki, Kazuyuki Aihara

**Affiliations:** 1 FIRST, Aihara Innovative Mathematical Modelling Project, Japan Science and Technology Agency, Meguro-ku, Tokyo, Japan; 2 Institute of Industrial Science, The University of Tokyo, Meguro-ku, Tokyo, Japan; 3 PRESTO, Japan Science and Technology Agency (JST), Kawaguchi, Saitama, Japan; Wake Forest School of Medicine, United States of America

## Abstract

Since the allocation of vaccines is often constrained by limited resources, designing an economical vaccination strategy is a fundamental goal of the epidemiological modelling. In this study, with the objective of reducing costs, we determine the optimal allocation of vaccines for a general class of infectious diseases that spread mainly via contact. We use an optimization routine to identify the roles of nodes with distinct degrees as depending on the cost of treatment to that of vaccination (*relative cost of treatment*). The optimal allocation drives vaccination priority to medium-degree nodes at a low relative cost of treatment or to high-degree nodes at a high relative cost of treatment. According to the presented results, we may adjust the vaccination priority in the face of an endemic situation.

## Introduction

Vaccines are often used to combat specific diseases, thereby preventing millions of deaths every year. Mathematical modelling of vaccine policies [1] and the human behavior that influences epidemic dynamics [2]–[7] have attracted considerable attention during the last few years. In order to control the outbreak of an infectious disease efficiently, a reasonable vaccination strategy is required, and its efficacy needs to be evaluated. To this end, the characteristics of an infectious disease often provide important guidelines for the determination of the optimal allocation of vaccines [8]. For instance, a strategy that takes into account the transmission rates of the influenza virus in different age groups and targets the group with the highest risk of infection, i.e., schoolchildren, has achieved the largest reduction in transmission [9], [10]. The efficacy of a vaccination strategy has often been evaluated by comparing it with other vaccination strategies that aim at reducing the transmission rate or infection risk [11]. However, a comparative analysis of different strategies is not sufficient for finding the optimal strategy [10]. Given certain outcome measures, such as the basic reproduction number [12], [13], and the morbidity and mortality rates of a disease [14], [15], and the economic considerations [16], the optimal allocation of vaccines can be determined accordingly using an optimization procedure [17], [18]. Since the available amount of vaccines is often limited, economic factors become fundamentally important. Thus, the requirement for a successful strategy would primarily involve the minimization of the total cost, and it might be more desirable to rely on the design of an economical vaccine policy.

For airborne diseases, such as the influenza virus, the assumption of a homogeneous mixing of the population is often reasonable for modeling the epidemic dynamics. However, for contagious diseases, such as sexually transmitted diseases (STDs), it may not be appropriate. This is because the sexual contact network within a population has been found to obey power-law degree distribution [19], wherein heterogeneity in the network leads to the epidemic process being characterized by new properties, such as the vanishing of the critical invasion threshold in the limit of infinite population [20], [21]. Consequently, heterogeneity leads to new intervention strategies [22]–[24], such as targeting vaccination at highly connected nodes [19].

Supposing that a fixed amount of vaccine is available, how to allocate it efficiently in the population is an essential issue with which public health officials are often concerned [25], [26]. Cost may be used to treat infected individuals, and it may be used to vaccinate susceptible individuals. The issue of minimizing the total costs of both treatment and vaccination, similar to the issue presented here, was explored in [16], where the network was assumed to be homogeneous, and thereby, nodes were equivalently treated. However, previous studies have shown that, depending on the detailed dynamics, nodes with distinct degrees play diverse roles. For instance, hub nodes play a crucial role in maintaining the static network robustness [24], whereas low-degree nodes are important for maintaining the dynamical network robustness [27].

In this paper, we are concerned with the role of nodes with distinct degrees in the design of an economical vaccination strategy. It may be useful to consider the factor of heterogeneity in different aspects of diseases, such as susceptibility, infectiousness, and latent and incubation periods; however, we focus particularly on the importance of contact. To achieve our objective of describing a general class of diseases that predominantly spread via contact, in this study we utilized a susceptible-infected-recovered (SIR) model and ignored side effects possibly caused by treatment, vaccination, and so on. The pursuit of the optimal allocation of vaccines that balances the trade-off between vaccination and treatment leads us to make the simple assumption that all infected individuals may receive treatment, and thus, the issue of the optimal ratio of individuals who receive treatment is beyond the scope of this paper. For further detailed analysis of this issue, please refer to Ref. [25]. In addition, in order to observe explicitly the relationship between vaccination and treatment, the endemic state of the disease in the long run is used, and consequently, this assumption constrains our study to the control of endemic situations and not to the control of emerging outbreaks.

In the study, we assume that a fixed amount of vaccines is provided at each time unit. Based on the epidemiological model that uses births and deaths, we investigate the optimal vaccination strategies in two cases: (i) homogeneous networks; (ii) heterogeneous networks. We find that in case (i), depending on the cost of treatment relative to that of vaccination (relative cost of treatment), optimal vaccination coverage varies between zero and the critical coverage required to eradicate the disease. In case (ii), it becomes almost impossible to derive the optimal allocation analytically, because the allocation of vaccine is uncertain and the number of variables involved in the model is increased. We use an optimization routine named *tabu search*, to obtain numerically the optimal allocations within a range of parameters. In the light of our findings, the optimal allocation is closely relevant to the relative cost of treatment. In the case of a low relative cost of treatment, vaccination priority usually goes to medium-degree nodes rather than high-degree nodes, which indicates that the role of high-degree nodes may have to be reevaluated; however, in the case of a high relative cost of treatment, vaccination priority may shift primarily toward high-degree nodes, where the reduction in the fraction of infected nodes crucially affects how much the total cost will be. This study may guide us to determine the optimal allocation of vaccines for a general class of infectious diseases that spread mainly via contact, and it may provide important insights into the role of nodes in the economical control of infectious disease, which will benefit public health services.

## Results

### Case of homogeneous networks

The theoretical understanding of the spread of epidemics is usually based on compartmental models, in which individuals in the population are classified into a discrete set of states and mixed homogeneously. In order to carry out the epidemiological analysis, the basic susceptible-infected-removed (SIR) model that uses births and deaths was employed, where the birth and death rates are assumed to be equal to 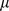. This model assumes that the time scale of an epidemic is longer than the demographic time scale. Unvaccinated susceptible individuals become infected by coming in contact with infectious individuals at the rate 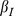, and infected individuals undergo recovery at rate 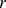. We assumed that a fixed vaccination coverage, 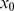, is provided at each time unit, even after the eradication of the disease. Since vaccines rarely provide full protection from diseases [28], partially effective (imperfect) vaccines are often used to protect both individuals and the entire population, as is the case for vaccines currently being developed against malaria [29] and the human immunodeficiency virus (HIV) [30]. Thus, the assumption of a partially effective vaccine was made in the model. The vaccine efficacy, 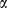, is captured by a reduced infection rate, 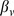, i.e., 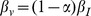 with 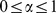, at which vaccinated individuals become infected.

Without economic considerations, the critical vaccination coverage, 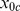, i.e., the fraction of the population that has to be vaccinated to avoid a major outbreak, should satisfy the condition 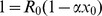, where 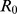 denotes the basic reproduction number, defined as 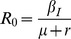 [31]. Thus, the critical vaccination coverage 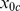 is derived as 
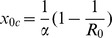
, indicating that 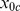 decreases with an increase in 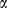 and increases with 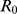. In particular, if 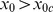, vaccination will lead to the eradication of the disease.

Let us consider the cost associated with the model. Suppose that the total cost is composed of two parts: The cost of vaccinating susceptible individuals and that of treating infected individuals. Obviously, vaccinating susceptible individuals will increase with the amount of vaccines used. For simplicity, we assume that the cost of vaccination exponentially depends on the vaccination coverage, as in Ref. [16], whereas the cost of treatment is proportional to the prevalence of the disease. In this study, discounting was ignored and the cost was counted only at the end of an epidemic [16]. Therefore, the total cost is defined as
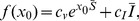
(1)where 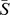 and 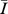 denote the stationary fractions of susceptible and infected nodes, respectively, and 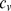 and 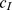 denote the per capita cost of vaccination and that of treatment, respectively. Typically, we have 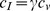 (where 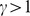 is referred to as the “relative cost of treatment”), indicating that the lower cost of vaccination would save us money as compared to the perceived high cost of treatment. The optimal solution to Eq. (1) is achieved by validating the first-order condition 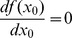 and given by
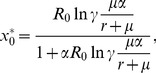
(2)where 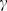 should satisfy the condition 
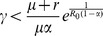
 for a meaningful solution to exist. 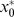 is a local minimal solution that is further confirmed by checking the second-order condition 
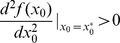
 (see Methods).

**Figure 1:**
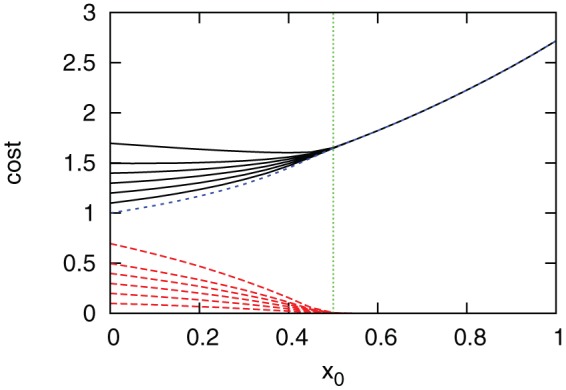
Cost versus 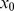
for 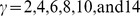 (from bottom to top) in homogeneous networks. (The green dotted vertical line) the theoretical solution 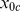; (the blue short dashed line) the cost of vaccination; (the red dashed lines) the cost of treatment; and (the black solid lines) the total cost. Parameters are set as 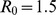, 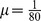, and 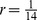. The basic per capita cost is set as 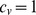 and the vaccine efficacy is 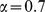.

Figure 1 shows the total cost (the black solid lines), the cost of treatment (the red dashed lines), and that of vaccination (the blue short dashed line) versus 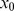. Obviously, the cost of treatment decreases with 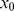, because the more available is the vaccination coverage, the smaller is the fraction of infected nodes at equilibrium. At 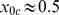, the outbreak is almost eradicated. If vaccines are continually provided, the cost of treatment decreases to zero, while the cost of vaccination increases continually (see the blue short dashed line in Fig. 1). Therefore, the total cost varies irregularly with the relative cost of treatment 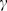, yielding a minimal solution 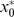 (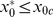).

**Figure 2:**
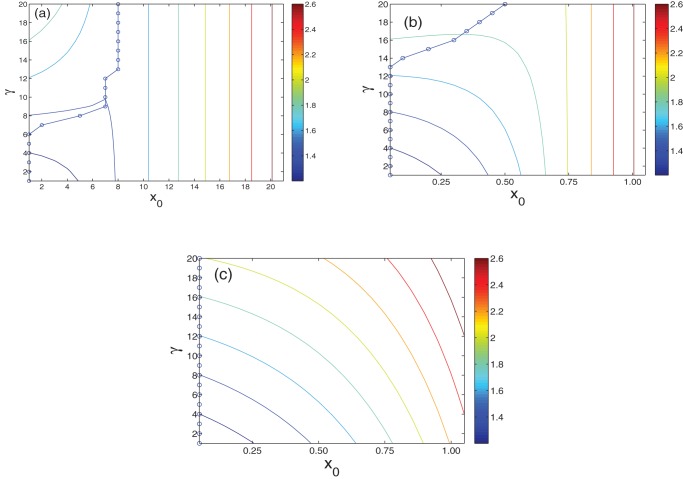
The total cost versus 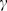 and 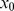 for different vaccine efficacy 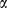. (a) 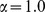; (b) 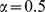; and (c) 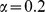. Circles indicate the optimal vaccine coverage 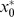 for each value of 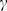. Other parameters are set to the same values as in Fig. 1.

The total cost as a function of 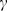 and 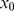 with regard to 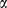 is shown in Fig. 2. The curves indicate that the optimal vaccination coverage is closely related to the relative cost of treatment 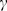 and the vaccine efficacy 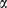. For example, if the vaccine efficacy is high (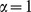) and the relative cost of treatment is low (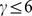), it is unnecessary to vaccinate any nodes at all; on the other hand, with an increase in 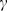, vaccines are required in order to reduce the prevalence of the epidemic (Fig. 2 (a)). With a reduction in 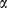, it would be more economical to stop vaccination (Fig. 2 (c)).

### Case of heterogeneous networks

In order to understand the role of nodes in the epidemic process with regard to vaccination, we investigated the optimal allocation of vaccines in heterogeneous networks, where the degree distribution follows power-law, i.e., 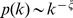 (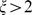) with a finite average connectivity 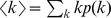. Since only a fraction 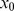 of the population at most can receive vaccines, achieving an efficient allocation at relatively low cost is an essential goal in the design of a public health strategy. Intuitively, a simple and direct design should account for nodes' degrees, that is, for each degree-block 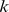, a fraction 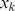 of the population with degree 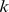 at most will receive vaccines. Therefore, the problem is to search the optimal allocation 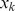 that minimizes the total cost subject to the constraint that the total consumption of vaccination is not more than the total resources, i.e., 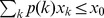.

Let us first investigate how the critical infection rate varies versus vaccine allocation in heterogeneous networks. With the mean-field approximation for each degree 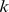 (see Methods), the effective infection rate 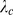 is given by
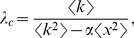
(3)which relates the network topology (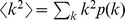) to the second moment of the vaccine allocation 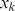, i.e., 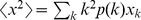. Equation (3) indicates that the critical infection rate 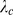 is determined by the network structure only before and after vaccination.

### Optimal allocation of vaccines in uncorrelated networks

In this study, instead of simply comparing a number of typical vaccination strategies, such as target [32], random [24], and acquaintance vaccination [23], we directly performed an optimization routine to determine the optimal allocation 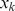. As demonstrated by previous efforts [32], targeting vaccination at hub nodes can efficiently eradicate the outbreak, but it is unknown if this strategy is still optimal in terms of economical considerations. In order to highlight the role of nodes with high degrees, nodes with degree larger than a certain critical value 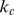 are stratified into one group, whereas nodes with the same degree 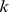 (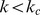), namely 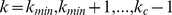, are stratified into the same groups. Consequently, nodes in the same group receive the same percentage of vaccines. For example, according to the degree distribution 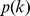, nodes can be stratified into eight groups as 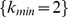, {3},...,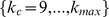, where 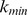 denotes the minimal degree and 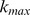 denotes the maximal degree. Subsequently, high-degree nodes are separated from the other nodes, and their role in the optimal allocation can be clarified.

The optimization problem of allocating vaccines in heterogeneous networks is described as
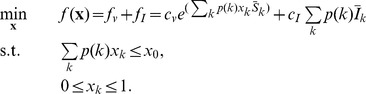
(4)


Obviously, the total number of vaccinated nodes 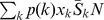 should not be greater than the total vaccine resources allocated to each degree 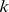, i.e., 
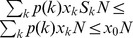
. 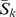 and 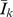 denote the fractions of susceptible and infected nodes at equilibrium, respectively. The objective function 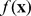 is composed of two components, the cost of vaccination, 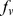, and that of treatment, 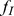, as seen in Eq. (4).

Using the tabu search [33], we determined the optimal allocation 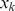 for the minimal cost 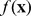 with regard to 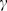. Intuitively, if the per capita cost of treatment 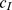 is high, more doses of vaccines are required to reduce the fraction of infected nodes, and vice versa. We performed the tabu search with 10 different initial solutions and chose the one with the lowest cost as the optimal solution.

We begin by testing networks generated using the uncorrelated configuration network model with a given degree distribution 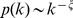 (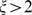) [34] (see Methods). This will produce the maximal degree 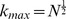, and the degree correlation is avoided. When networks have been generated, the conditional probability 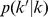 that a node with degree 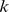 is connected to a node with degree 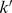 is determined. Due to the uncertainty in the allocation of vaccines to the nodes with degree 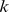, 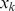, it is difficult to derive the explicit forms of 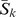 and 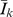 at equilibrium in the present model. Thus, we numerically calculate them with the model in Eqs. (10) and (11) (see Methods).

**Figure 3:**
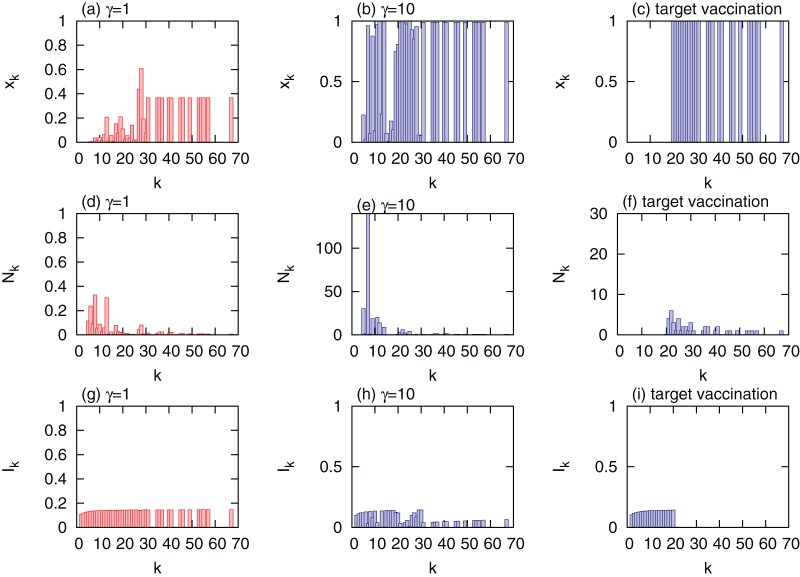
Comparison of optimal allocations of vaccines with target vaccination in uncorrelated scale-free networks. Optimal allocation 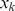 (a), 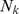 (d), and 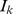 (g) for 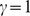 (left column); optimal allocation 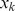 (b), 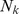 (e), and 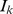 (h) for 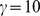 (center column); target allocation 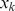 (c), 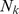 (f), and 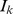 (i) (right column). At equilibrium, the fractions of infected nodes are 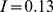 (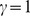), 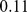 (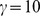), and 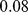 (target vaccination), respectively. The total costs for the optimal allocation are 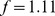 (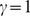), 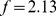 (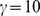), 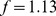 (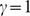 for target vaccination), and 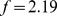 (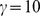 for target vaccination). 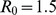 and the maximal vaccination coverage is 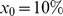. The degree distribution is generated with 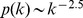 and the network size 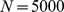. The minimal degree is 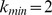 and the maximal degree is 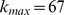. Nodes are grouped into 30 groups.

In order to understand the impact of the relative cost of treatment 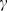 on the optimal allocation 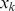, we performed the optimization routine for a low relative cost of treatment (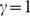) and high relative cost of treatment (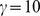), respectively. Without loss of generality, in the following, vaccines are assumed to be perfect, that is, 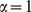. Moreover, to understand the characteristics that 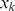 possesses, we recorded the distribution of vaccinated nodes for each degree 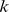, 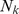, given by 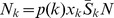, and the distribution of the fraction of infected nodes at equilibrium, 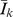. Comparing the optimal allocations 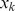 with regard to 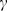, we found a significant difference between them. For a low relative cost of treatment (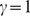), the optimal allocation vaccinates fewer nodes with extremely high degrees and low degrees (Fig. 3 (a) and (d)). For example, only 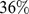 of nodes with degree larger than 30 are vaccinated, and nodes with degree less than 10 are not vaccinated. The result suggests that, for low relative cost of treatment, solely targeting vaccination at high-degree nodes may be considerably less economical.

With an increase in the relative cost of treatment (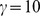, Fig. 3, center column), more high-degree nodes are vaccinated, e.g., most nodes with degree larger than 20 are vaccinated. This implies that, for the high relative cost considered in this analysis, targeting vaccination at high-degree nodes can efficiently reduce the fraction of infected nodes at equilibrium and the perceived cost of treatment as well. To understand the characteristics of the optimal allocation further, we compared them with those of target vaccination (Fig. 3, right column). Target vaccination is carried out by vaccinating all nodes with degree larger than some value (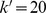). Properties, such as the distribution of vaccinated nodes, 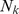, and the distribution of the fraction of infected nodes, 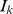, are also shown in Fig. 3. A comparison of the optimal vaccination for 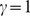 and 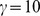 with target vaccination, revealed that when the relative cost of treatment is low, optimal allocation performs much better than target vaccination; when the relative cost of treatment is high, target vaccination performs nearly identically to optimal allocation. In the latter case, the reduction of infected nodes becomes crucial to that of the total cost. This is confirmed in Fig. 3 (h), where fewer nodes are infected at equilibrium. Since, in practice, detailed knowledge of the optimal allocation is not often available in advance, targeting vaccination at high-degree nodes may be taken as an alternative strategy in this case.

The results presented above suggest that the design of an optimal allocation should take into account factors that include the relative cost of treatment. From a realistic point of view, it provides us with some insights into the design of an economical allocation of vaccines for the control of diseases that spread via contact. These diseases are typically characterized by sexually transmitted diseases (STDs), such as gonorrhea, chlamydia, and syphilis, where individuals are infected through sexual contact with an infectious person. Since the human sexual contact network follows power-law distribution [35], in light of our results, we speculate that if the cost of treating STDs is extremely high, people who have more sexual partners (hub individuals) should be prioritized for vaccination. Thus, the perceived high cost of treatment caused by the potential infection of hub individuals may be reduced. To this end, tracing individuals' sexual contacts to obtain precise knowledge concerning the people who are well connected in the sexual network, as was done in the Swedish survey of sexual behavior in 1996 [19] is a fundamental requirement [36]. If the cost of treatment is not very high, precise knowledge of personal contacts may not be required.

### Optimal allocation of vaccines in correlated networks

The results presented above are based on the configuration network model where degree correlation is omitted; however, in the real world, networks often show some level of degree correlations [37]. The degree correlation of a network can be quantified by the assortativity [37]

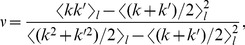
(5)where 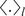 denotes the average over all links and 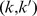 denotes the degrees of the two nodes at either end of the links. A positive (assortative) or negative (disassortative) degree correlation is denoted by the sign of 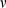. An alternative method of calculating degree correlation is to measure the nearest neighbor degree 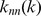, which is an increasing (decreasing) function of 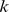 for networks with a positive (negative) correlation. To generate networks with a desired degree correlation, we exchanged the end points of two edges chosen at random in uncorrelated networks until the desired degree correlation was achieved.

**Figure 4:**
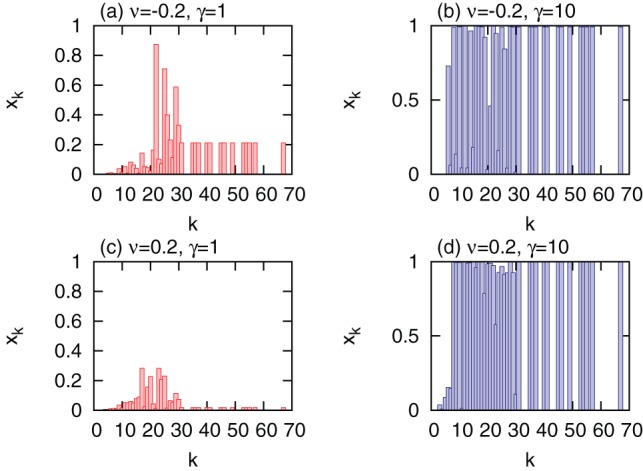
Comparison of optimal allocations of vaccines 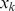
in networks with different degree correlations 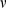. (a) 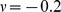 and 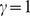; (b) 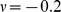 and 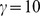; (c) 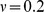 and 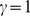; (d) 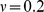 and 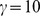. 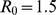 and the maximal vaccination coverage is 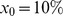. Other parameters are set to the same values as in Fig. 3. Nodes are grouped into 30 groups.

Optimal allocation of vaccines was then implemented on networks with typically positive and negative degree correlations (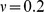 and 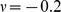, respectively); however, other parameters, such as network size and the average connectivity were the same as in uncorrelated networks, see Fig. 4. Irrespective of the degree correlations, we obtained results that are qualitatively similar to those on networks with no degree correlation. The findings represent an advanced step toward the understanding of the role of high-degree nodes in the design of an economical vaccine allocation. Depending on the relative cost of treatment, the role of high-degree nodes is particularly different, driving the optimal vaccination strategy into two scenarios. When the relative cost of treatment is low, the tradeoff between the costs of vaccination and treatment drives the system to vaccinate fewer nodes with high degrees. When the relative cost of treatment is high, primarily the fraction of infected nodes determines how much the total cost will be. Under this condition, prioritizing the vaccination of high-degree nodes is crucial to the reduction of the fraction of infected nodes and of the total cost as well.

## Discussion

In this study, we used a general mathematical model to evaluate the optimal allocation of vaccines by focusing on a heterogeneous contact structure and accounting for economic factors. In this framework, for homogeneous networks, the optimal vaccination coverage with minimal cost varies between zero and the critical coverage, depending on the relative cost of treatment, as well as the vaccine efficacy.

The epidemic model (SIR) we used is very simple, no heterogeneity related to diseases being considered, but our preliminary analysis of heterogeneity in contact clarifies the role of nodes with different degrees in the optimal allocation of vaccines. In heterogeneous networks, depending on the relative cost of treatment, the optimal allocation varies such that the tradeoff between the cost of vaccination and that of treatment is balanced. In the case of low relative cost of treatment, target vaccination may have been overestimated in the present model. With an increase in the relative cost of treatment, vaccination priority may shift toward nodes with high degrees. The comparison analysis shows that the optimal allocation is superior to target vaccination in terms of minimal total cost but not in terms of minimal prevalence, whereas target vaccination is superior to the optimal allocation in terms of reducing the prevalence but not of minimizing the cost.

For the definition of cost, following Ref. [16], we assumed that the cost of vaccination is exponentially dependent on the ratio of vaccinated individuals, whereas the cost of treatment is linearly dependent on the ratio of treated individuals. It might be useful to consider disease-related costs, such as those of side effects of treatment, morbidity, mortality, and loss of productivity, which are related to details of a specific disease.

The results of our present study are particularly useful for guiding the optimal allocation of vaccines for diseases that predominantly spread via contact, i.e., STDs, where heterogeneity in contact dominates the epidemic dynamics. In the real world, the main aim of the public health policy is usually to minimize the outbreak of an infectious disease. These results may provide insights for planning future cost-aware strategies for multi-objective optimization of vaccination, such as simultaneously optimizing both the cost and prevalence, which will be the primary concern of public health officials.

Overall, our study demonstrates that the design of an economical allocation of vaccinations should incorporate a number of important factors involved in the model. It may be possible to achieve better control of STDs if advances in our understanding of the nodes' role in epidemiology and transmission dynamics can be integrated into future intervention strategies.

## Methods

### Derivation in homogeneous networks

We apply the mass-action assumption, where all individuals have the same contact rate, to obtain the epidemic dynamics for the SIR model with imperfect vaccines [6],
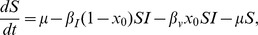


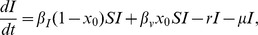


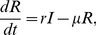
(6)where 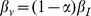 and 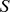, 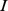, and 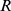 denote the proportions of susceptible, infected, and recovered individuals, respectively. In particular, 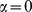 represents the case of the classic model without vaccination, and 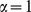 represents the case of perfect vaccines, where all vaccinated individuals obtain complete protection from infection. In the case where no vaccines are given, the basic reproductive number is 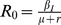. The fractions of susceptible and infected individuals at equilibrium are subsequently given by



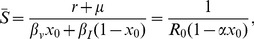
(7)

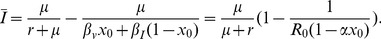
(8)The total cost is composed of two parts: The cost of vaccinating susceptible individuals and the cost of treating infected individuals. Therefore, the total cost is written as
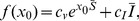
(9)


where 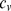 and 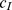 denote the per capita cost of vaccination and that of treatment, respectively. By inserting Eqs. (7) and (8) into (9), the optimal solution 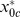 can be derived as shown in Eq. (2).

### Derivation of effective infection rate in uncorrelated networks

In order to investigate the effective infection rate 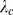 when vaccination is induced, we consider the time evolution of the magnitudes of 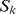, 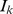, and 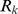, which denote the densities of the susceptible, infected, and recovered vertices of degree 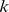, respectively. These variables are connected by means of the normalization condition, and at the mean-field level, they satisfy the following set of coupled differential equations [20], [21]

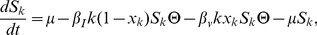
(10)

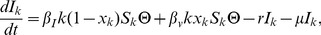
(11)

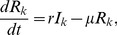
(12)where 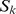, 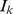, and 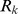 satisfy 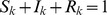. 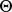 indicates the probability that a vertex is reached by following a randomly chosen link whose end node is infected, written as
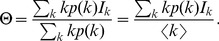
(13)When stationary conditions are imposed, the equilibrium of the model is given by



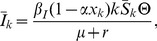
(14)

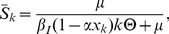
(15)

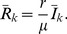
(16)


By inserting Eq. (14) into Eq. (13), we have
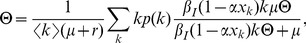
(17)where 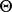 is a function of itself, and 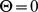 is always a trivial solution. If there exists a nonzero solution, the condition



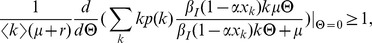



should be satisfied, which is rewritten as
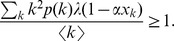



Then, the effective infection rate 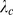 is derived as
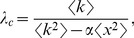
(18)which indicates that below 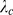 the disease is eradicated from the network, and above it there is an endemic state. In addition, 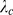 depends on the first (second) moment of the degree distribution, the second moment of the vaccination distribution, and the vaccine efficacy 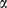. If there are no available vaccines, i.e., 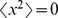, with 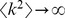, 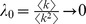, indicating that any disease can spread in heterogeneous networks with the degree distribution 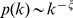 (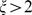). With the introduction of vaccines, 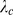 transforms to an explicit function in the form of 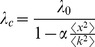. In real-world networks, with a finite value of 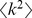, 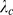 increases with the vaccine efficacy 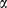, and thus, highly efficient vaccines may halt the spread of an infectious disease.

### Derivation of optimal allocation of vaccines in uncorrelated networks

It should be noted that 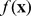 cannot be explicitly defined because it is not easy to solve 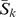 and 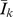 analytically due to the complexity of heterogeneity in the network structure and the uncertainty of vaccine allocation in each block 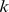, 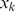. Thus, we numerically solve 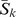 and 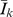 using Eqs. (10) and (11). In order to solve the optimization problem, a heuristic algorithm named tabu search is subsequently implemented [33], [38]–[41] with 10 different initial solutions. Then, we choose the solution with the minimal cost among the 10 runs as the optimal solution. Finally, the optimal solution is further improved by taking the optimal solution previously obtained as an initial solution to start the tabu search again.

The processes of the tabu search for solving Eq. (4) are described as:

- Step 1: Generate an initial feasible vector 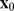 that satisfies the inequality constraints, and set the optimal solution 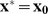, and calculate the optimal cost value 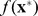. Set the time step 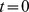.
- Step 2: Stop and output the optimal solution 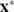 and 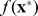 if a prescribed condition is satisfied; otherwise, generate a random vector 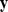 that is feasible, and calculate the total cost 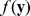.
- Step 3: Update the optimal solution if 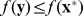 as 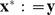. If 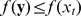 or if 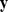 does not satisfy the tabu conditions, set 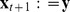; else set 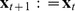. Set 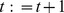 and return to step 2.

The condition 
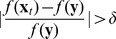
, where 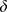 denotes the ratio of improvement or destruction that will be accepted if the new move is accepted, is used to determine whether a move is tabu. Thus, the new solution 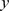 in Step 2 is assumed tabu if the ratio of the total change in the objective function is larger than 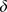. The terminal condition is that the present step reaches the predefined maximal number of iteration steps. Here, the maximal number of iteration steps is set as 2000.

### Configuration network models

In order to avoid degree correlation, the substrate networks are generated using the configuration model based on the Molloy-Reed algorithm [34]. Each vertex 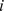 is assigned a degree 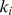 from a given degree distribution 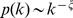
(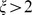), subject to the constraint 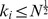, where 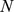 indicates the network size.

### Sensitivity analyses

**Figure 5:**
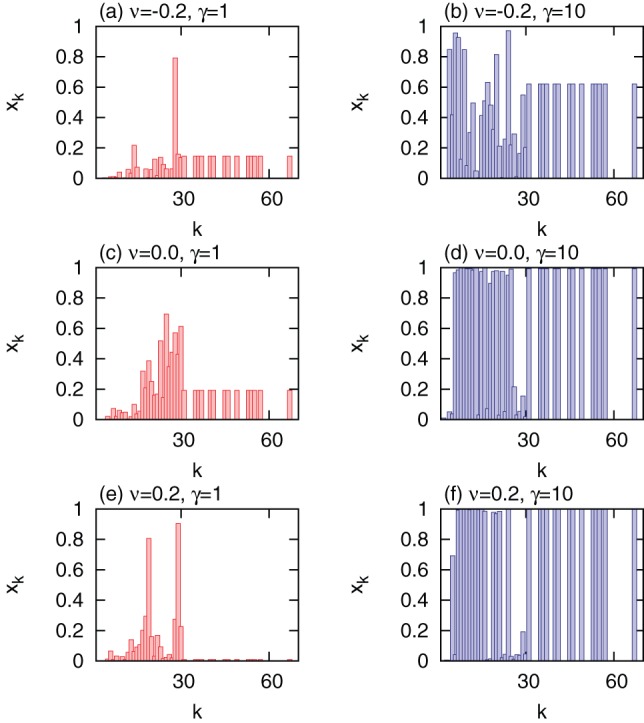
Optimal allocations of vaccines 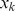
in correlated scale-free networks with different degree correlations 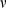
for 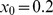. (a) 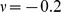 and 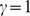; (b) 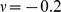 and 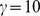; (c) 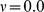 and 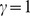; (d) 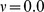 and 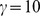; (e) 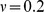 and 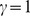; (f) 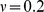 and 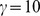. Other parameters are set to the same values as in Fig. 3. Nodes are grouped into 30 groups.

**Figure 6:**
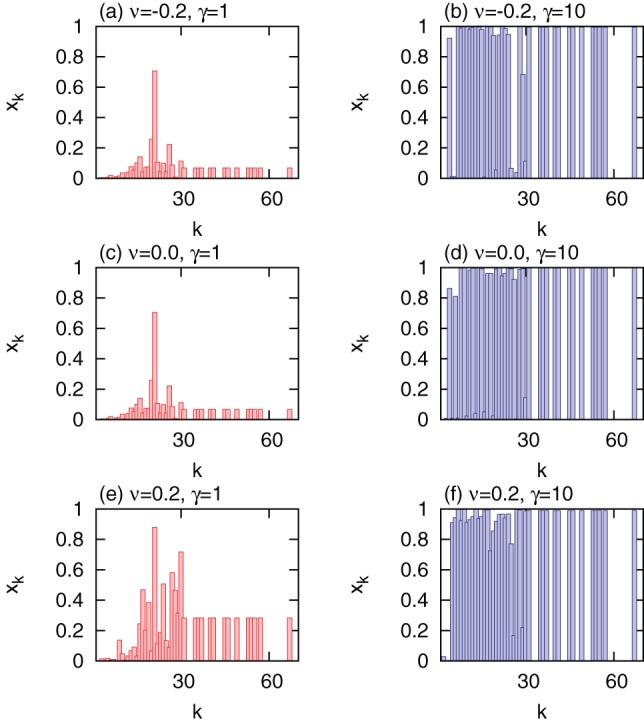
Optimal allocations of vaccines 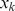
in correlated scale-free networks with different degree correlations 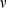 for 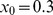. (a) 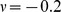 and 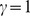; (b) 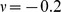 and 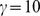; (c) 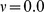 and 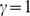; (d) 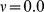 and 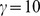; (e) 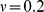 and 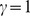; (f) 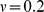 and 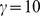. Other parameters are set to the same values as in Fig. 3. Nodes are grouped into 30 groups.

To explore the robustness of the results, we conducted sensitivity analyses with varied vaccination coverage 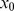 ranging from 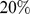 to 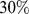, while other parameters remained the same as given in the main text (without loss of generality, 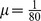 and 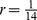 throughout this paper. We also tested different choices of epidemiological parameters 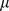 and 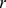 and obtained qualitatively similar results.) Figures 5 and 6 show that even in correlated scale-free networks, with the assumed vaccination coverage 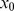 that we tested, results that are qualitatively similar to those in the main text were obtained, i.e., with a low relative cost of treatment, fewer nodes with high degrees are vaccinated, whereas with a high relative cost of treatment, more nodes with high degrees are vaccinated. Therefore, irrespective of the vaccination coverage, the optimal allocations of vaccines show qualitatively similar properties.
